# Pan-Immune-Inflammatory Value Predicts the 3 Months Outcome in Acute Ischemic Stroke Patients after Intravenous Thrombolysis

**DOI:** 10.2174/0115672026276427231024045957

**Published:** 2023-12-30

**Authors:** Shan Wang, Lulu Zhang, Huan Qi, Lulu Zhang(F), Qi Fang, Lanfeng Qiu

**Affiliations:** 1 Department of Neurology, The First Affiliated Hospital of Soochow University, Suzhou, China;; 2 Department of General Medicine, The First Affiliated Hospital of Soochow University, Suzhou, China;; 3 Department of Neurology, Dushu Lake Hospital Affiliated to Soochow University, Suzhou, China;; 4 Department of Emergency, The First Affiliated Hospital of Soochow University, Suzhou, China

**Keywords:** Pan-immune-inflammation value, acute ischemic stroke, intravenous thrombolysis, clinical outcome, multivariate logistic regression analysis, new inflammation index

## Abstract

**Background and Purpose:**

Immune and inflammatory response plays a central role in the clinical outcomes of stroke. This study is aimed to explore the clinical significance of the new inflammation index named pan-immune-inflammation value (PIV) in patients with acute ischemic stroke (AIS) after intravenous thrombolysis therapy (IVT).

**Methods:**

Data were collected from 717 patients who received IVT at the First Affiliated Hospital of Soochow University. Baseline data were collected before intravenous thrombolysis. Multivariate logistic regression analysis was used to assess the association between PIV and 3 months clinical outcome after intravenous thrombolysis. We also used receiver operating characteristic (ROC) curves analysis to assess the discriminative ability of PIV, platelet to lymphocyte ratio (PLR), neutrophil to lymphocyte ratio (NLR), and systemic immune-inflammation index (SII) in predicting 3 months poor outcome.

**Results:**

Of 717 patients, 182 (25.4%) patients had poor outcomes at 3 months. Patients with 3 months of poor outcome had significantly higher PIV levels compared to those with favorable outcomes [316.32 (187.42-585.67) *vs*. 223.80 (131.76-394.97), *p* < 0.001)]. After adjusting for potential confounders, the risk of 3 months of poor outcome was significantly higher among patients whose PIV fell in the third quartile (244.21-434.49) and the fourth quartile (> 434.49) than those in the first quartile (< 139.93) (OR = 1.905, 95% CI: 1.040-3.489; OR = 2.229, 95%CI: 1.229-4.044). The area under the ROC curve of PIV to predict 3 months of poor outcome was 0.607 (95%CI: 0.560-0.654; *p* < 0.001). The optimal cut-off values of PIV were 283.84 (59% sensitivity and 62% specificity).

**Conclusion:**

The higher levels of PIV were independently associated with 3 months of poor outcomes in AIS patients receiving IVT. PIV like other inflammatory factors (PLR, NLR, and SII), can also predict adverse outcomes after IVT in AIS patients.

## 1 INTRODUCTION

According to the latest Global Burden of Disease Study report, stroke is still the second leading cause of death worldwide, accounting for 11.6% of the total number of deaths, of which ischemic stroke accounts for 62.4% of the total number of strokes [1]. Although intravenous thrombolysis therapy (IVT) can reduce neurological deficits in patients with acute ischemic stroke, stroke is still the third leading cause of disability worldwide [2]. The identification of biomarkers that can predict the poor outcome of ischemic stroke can help to give these high-risk patients targeted care in advance and provide more comprehensive medical measures to improve their prognosis.

Recent studies are increasingly exploring the effects of immune-inflammatory mechanisms on clinical outcomes after stroke [3]. Inflammatory indicators such as systemic immune-inflammation index (SII), platelet-lymphocyte ratio (PLR), and neutrophil-to-lymphocyte ratio (NLR) have recently been reported to be able to predict clinical outcomes in patients with AIS received IVT [4, 5]. A novel inflammatory biomarker called pan-immune-inflammation value (PIV) integrates neutrophil, monocyte, platelet, and lymphocyte counts to reflect a more comprehensive status of the systemic immune inflammatory response [6]. Compared with SII and PLR, recent studies have found that PIV is a better predictor of mortality in ST-elevation myocardial infarction patients [7].

To our knowledge, the clinical significance of PIV in AIS patients after thrombolysis therapy has not been reported. Therefore, this study is designed to explore the relationship between PIV and 3-month clinical outcomes of the patients who received IVT after ischemic stroke.

## 2 MATERIALS AND METHODS

### 2.1 Patients

We retrospectively collected the details of 812 patients with acute ischemic stroke treated by IVT from January 2017 to August 2022 in the emergency green channel of the First Affiliated Hospital of Soochow University. All patients received intravenous thrombolysis therapy within 4.5 hours of onset. Furthermore, we excluded 46 of the 812 patients who suffered from severe inflammation or infectious diseases, 17 with incomplete baseline data of admission, and 32 who failed to follow up after 3 months. These exclusions resulted in 717 patients finally being included in this study. The flow diagram of the study is given in Fig. (**1**). Ethical approval for this study was obtained from the ethics committees of our hospital.

### 2.2 Intravenous rt-PA Thrombolysis Therapy

The standard dosage was 0.9 mg per kilogram of body weight (10% as a bolus for 1 min and remaining 90% as an infusion for 1 hour; maximum dose, 90 mg). The low dosage was 0.6 mg per kilogram of body weight (15% as a bolus for 1 min and remaining 85% as an infusion for 1 hour; maximum dose, 60 mg). All patients received only one dose.

### 2.3 Pan-Immune-Inflammation Value Calculation

PIV = [neutrophil count (10^3^/mmc) × platelet count (10^3^/mmc) × monocyte count (10^3^/mmc)] / lymphocyte count (10^3^/mmc).

SII = [neutrophil count (10^3^/mmc) × platelet count (10^3^/mmc)] / lymphocyte count (10^3^/mmc).

NLR = neutrophil count (10^3^/mmc) / lymphocyte count (10^3^/mmc).

PLR = platelet count (10^3^/mmc) / lymphocyte count (10^3^/mmc).

### 2.4 Data Collection

The basic information includes age, sex, smoking and drinking history, and antithrombotic medication history (anti-platelet agents or any type of oral anticoagulants). Baseline data at admission include blood pressure, blood glucose, National Institute of Health stroke scale (NIHSS) score, onset to treatment (OTT) time, laboratory data, and Trial of Org 10172 in Acute Stroke Treatment (TOAST) classification. NIHSS score was used by professional neurologists to assess the severity of stroke for all patients at admission. Cigarette smoking was defined as smoking at least one cigarette a day for more than six months [8]. Alcohol consumption was defined as consuming at least 1 alcoholic drink every day during the last year [9]. Hypertension was defined as the history of taking any type oral anti-hypertensive drugs or previous diagnosis of hypertension or blood pressure higher than 140/90mmHg during hospitalization [10]. Diabetes mellitus was defined as previously diagnosed with taking oral hypoglycemic drugs or diabetes diagnosed during hospitalization [10]. Atrial fibrillation was defined as previously diagnosed or a clinical diagnosis of atrial fibrillation during hospitalization. Anti-thrombotic was defined as the regular administration of anti-platelet agents or any type of oral anticoagulants before admission [11]. Dyslipidemia was defined as any kind of dyslipidemia previously diagnosed or at least having one of the following findings including increased total cholesterol [≥ 240 mg/dL (6.20 mmol/L)], LDL-C [> 160 mg/dL (4.13 mmol/L)], or triglyceride levels [> 200 mg/dL (2.25 mmol/L)] or decreased HDL-C [< 40 mg/dL (1.03 mmol/L)] during hospitalization [12]. Previous stroke was defined as having a history of transient ischemic stroke, ischemic stroke, intracerebral hemorrhage, or subarachnoid hemorrhage [10]. Follow-up data after three months were obtained by trained nurses through telephone consultation. We used the modified Rankin Scale (mRS) to assess the recovery of patients' neurological function at 3 months which scores ≥ 3 were poor outcomes and scores ≤ 2 were favorable outcomes.

### 2.5 Statistical Analysis

We applied SPSS version 21.0 (SPSS, Inc., Chicago, IL, USA) to analyze the collected data. Variables of normal distribution were expressed as mean ± standard deviation with the sample *t*-test. Variables of non-normal distribution were expressed as median (interquartile range) with the Mann-Whitney *U* test. Categorical variables were presented as percentages. The Chi-square test or Fisher’s exact probabilities test was used for categorical variables. We divided the calculated PIV level into quartiles, and used the first quartile value as a reference for multivariate logical regression analysis to detect whether the PIV level is independently related to prognosis after three months. Variables with *p* < 0.05 in univariate analysis were included as the main covariates in the binary logistic regression model. We used variance inflation factors (VIF) to examine multicollinearity and significant interactions between independent variables. The independent variables with multicollinearity relations (VIF>5) were eliminated in multivariate logical regression analysis. In this study, the receiver operating curve (ROC) was applied to analyze the accuracy of the prognosis of PIV, PLR, NLR and SII for the 3-month outcome of AIS patients receiving thrombolysis. The differences in discriminative ability were tested using the DeLong method and use R version 4.0.3 software to draw the corresponding figure. A *p* < 0.05 was considered statistically significant.

## 3 RESULTS

### 3.1 Baseline Characteristics

After a series of exclusions, a total of 717 patients were observed. The average age of patients was 68 (58-75) years old, of which 485 (67.6%) patients were male. The median NIHSS score on admission was 6 (3-11). The media PIV level was 244.21 (139.93-434.49) shown in Table **1**.

### 3.2 Patient Characteristics Between Favorable Outcome Group and Poor Outcome Group

The baseline characteristics between favorable outcome group and poor outcome group were provided in Table **1**. Of 717 patients, 182 had poor outcome and 535 had favorable outcome at 3 months. Patients with favorable outcome were usually younger [66 (57-74) *vs*. 73(65-80), *p* < 0.00^1^], lower rate of diabetes, atrial fibrillation, history of stroke [123(23.0%) *vs*. 65(35.7%), *p* = 0.001; 93 (17.4%) *vs*. 66 (36.3%), *p* < 0.001; 83(15.5%) *vs*. 44(24.2%), *p* = 0.0^1^]; lower levels of total bilirubin (TBIL) [16.4 (12.6-21.8) *vs*. 18.2 (14.5-26.3), *p* < 0.00^1^], glucose [6.82 (5.73-8.69) *vs*. 7.48 (6.23-10.24), *p* < 0.00^1^], white blood cells [7.47 (6.29-9.26) *vs*. 7.98 (6.56-10.04), *p* = 0.0^34^], neutrophils [4.91 (3.80-6.67) *vs.* 5.86 (4.22-7.93), *p* < 0.00^1^], high-density lipoprotein (HDL) [1.01 (0.86-1.20) *vs*. 1.09 (0.88-1.32), *p* = 0.00^9^]; higher levers of lymphocytes [1.73 (1.25-2.27) *vs*. 1.46 (1.05-1.90), *p* < 0.00^1^], triglyceride (TG) [1.29 (0.96-1.77) *vs*. 1.14 (0.92-1.60), *p* = 0.00^8^]. There was a statistically significant difference in NIHSS scores on admission [4 (2-8) *vs*. 12 (8-18), *p* < 0.00^1^] and TOAST classification (*p* < 0.001) between favorable outcome group and poor outcome group. Compared to the patients in the poor outcome group, the patients in the favorable outcome group possessed lower levels of SII [558.01 (345.60-907.26) vs. 738.76 (488.59-1270.92), *p* < 0.00^1^], PLR [115.38 (84.82-167.60) *vs*. 132.76 (96.65-173.35), *p* = 0.0^10^], NLR [2.87 (1.81-4.76) *vs*. 3.87 (2.39-6.56), *p* < 0.00^1^] and PIV [223.80 (131.76-394.97) *vs*. 316.32 (187.42-585.67), *p* < 0.00^1^]. Furthermore, between the two groups, PIV stratified after quartering was still notably different (*p* < 0.001). The distribution of PIV levels in the two groups was shown in Fig. (**2**).

### 3.3 Association Between Baseline Characteristics and Clinical Outcome in Multivariate Logistic Regression Analysis

PIV was converted into grade variables in multivariate logistic regression analysis. After adjusting for all confounders except for VIF > 5, PIV level was positively correlated with 3 months poor prognosis. Additionally, in Table **2**, patients in the fourth PIV quartile indicated a notable difference with OR of 2.229 (95%CI: 1.229-4.044, *p* = 0.008) compared with those in the first quartile. Age (OR = 1.022, 95% CI: 1.001-1.043, *p* = 0.036), NIHSS score (OR = 1.200, 95% CI: 1.154-1.249, *p* < 0.001), diabetes mellitus (OR = 3.333, 95% CI: 1.911-5.813, *p* < 0.001), HDL (OR = 2.094, 95% CI: 1.038-4.224, *p* = 0.039), history of stroke (OR = 1.705, 95% CI: 1.029-2.825, *p* = 0.038) and TOAST classification were also independently associated with 3-month prognosis in Table **2**.

### 3.4 Receiver Operating Characteristic Curve Analysis for 3-Month Outcome

Receiver Operating Characteristic (ROC) curve analyses were performed to compare the predictive performances among PIV and other popular inflammatory indexes, such as SII, PLR and NLR in Table **3** and Fig. (**3**). According to the ROC curve analysis, the optimal cut-off value of PIV that best discriminated poor outcome was 283.84 (59% sensitivity and 62% specificity). There was no significant difference in efficiency between PIV and other popular inflammatory indexes for predicting poor outcomes (PIV *vs*. SII: 0.607 *vs* 0.598, *p* = 0.478; PIV *vs*. PLR: 0.607 *vs*. 0.564, *p* = 0.059; PIV *vs.* NLR: 0.607 *vs* 0.617, *p* = 0.541).

## 4 DISCUSSION

In this study, we found that the higher the PIV level, the higher the risk of 3 months poor outcome in AIS patients after IVT. Through multivariate regression analyses, PIV level was significantly correlated with 3 months poor outcome of patients receiving thrombolysis. The ROC curve showed that PIV had a similar predictive ability for the 3 months poor outcome after thrombolysis compared to SII, PLR and NLR. There was no significant difference in their predictive ability. To our best knowledge, this is the first study to investigate the association between PIV and the 3 months clinical outcomes of AIS patients receiving IVT.

After cerebral ischemia injury, the damage-associated molecular patterns (DAMPs) released by necrotic cells activate resident immune cells in the central nervous system, such as microglia and astrocytes, which subsequently attract peripheral immune cells to activate adaptive immune responses [13]. The activated immune system along with the deactivated neuroendocrine and autonomic nervous systems, link the center with the periphery, leading to the systemic immune inflammatory response [14]. There is increasing evidence to suggest that the global immune inflammatory response can affect the clinical outcomes of stroke [15, 16]. Liu *et al.* reported that patients with poor prognosis after intravenous thrombolysis had higher NLR values [17]. In 2022, studies found that SII was closely related to the short (90-day) and long (1-year) term prognosis of patients with acute ischemic stroke, and patients with higher SIIs were more likely to have poor outcomes [18]. In another study, Xu *et al.* found that patients with unfavorable outcomes had significantly higher PLR than those with favorable outcomes, and the PLR values of the patients who died at 3 months were higher than those of the surviving patients [19]. What’s more, Gong *et al.* discovered that PLR and NLR was associated with post-thrombolysis early neurological deterioration [5]. Our study revealed that 3 months poor outcome group had significantly higher PLR, NLR and SII values compared to the group with favorable outcome. In addition, we found that PIV levels were elevated in the poor outcome group and determined that the high level of PIV was an independent risk factor for poor outcome at 3 months after IVT in AIS patients.

Fuca *et al*. identified PIV as a new Immune-inflammatory biomarker in patients with metastatic colorectal cancer (mCRC), and PIV had a stronger predictor of survival outcomes in first-line therapy mCRC patients than SII and PLR [6]. A systematic review evaluated the association between survival and PIV in cancer (colorectal cancer, melanoma, breast cancer, and non-small cell lung cancer), the results showed that patients with higher PIV levels had a significantly higher risk of death than patients with lower PIV levels, and the risk of disease progression or death was increased in patients with higher PIV levels [20]. In cardiovascular disease, Murat *et al.* found that PIV was better than PLR and SII in predicting one-year and one-month all-cause mortality in STEMI patients [7]. The Immune and inflammatory response is a common process in the clinical manifestations of cardiac and cerebral acute ischemia following atherothrombosis [21]. Therefore, PIV as a more systematic inflammation index, should also be considered when assessing the effect of inflammation on the clinical outcomes of AIS patients after IVT.

Previous studies showed that high SII, NLR and PLR are independent predictors of the independent risk factors for poor prognosis at 3 months of AIS patients [4, 22, 23]. In this study, we found that PIV was not inferior to SII, NLR, or PLR in predicting the prognosis of ischemic stroke, but not superior to any of them. This may be because the PIV data in this study came from laboratory data at the time of admission. It has been confirmed that the predictive power of inflammation indicators 24 hours after thrombolytic therapy was stronger than that at admission [24]. Compared to SII, NLR and PLR, PIV includes not only lymphocyte and platelet, but also monocyte and neutrophil, suggesting that PIV is a more systemic indicator of inflammation. However, the immune inflammatory response in ischemic stroke is a dynamic process [25]. This may have contributed to the fact that PIV at admission in this study did not show a significant advantage compared to other traditional biomarkers.

This study still has several potential limitations. First, this is a retrospective study from a single center, and the results are limited by the sample size and study population. Secondly, some risk factors that may be associated with poor outcome after thrombolysis in ischemic stroke patients, such as hyperhomocysteinemia [11] and plasma high mobility group box protein 1 [26], we were not able to capture and further analyze such potential factors. Third, PIV from AIS patients after thrombolysis was not further analyzed in this study, and whether changes in PIV before and after thrombolysis are more valuable needs further investigation. Moreover, previous studies have shown that higher levels of inflammation are associated with an increased incidence of stroke-associated pneumonia and post-stroke depression [27-29], and the long-term prognosis of PIV after stroke should be further studied. Despite these limitations mentioned above, this study is the first to report the relationship between PIV levels and clinical outcome in patients with acute ischemic stroke after intravenous thrombolysis.

## CONCLUSION

The higher levels of PIV were independently associated with poor outcome in AIS patients receiving thrombolysis. PIV had a similar predictive ability compared with popular biomarkers like PLR, NLR and SII for 3-month poor outcome.

## Figures and Tables

**Fig. (1):**
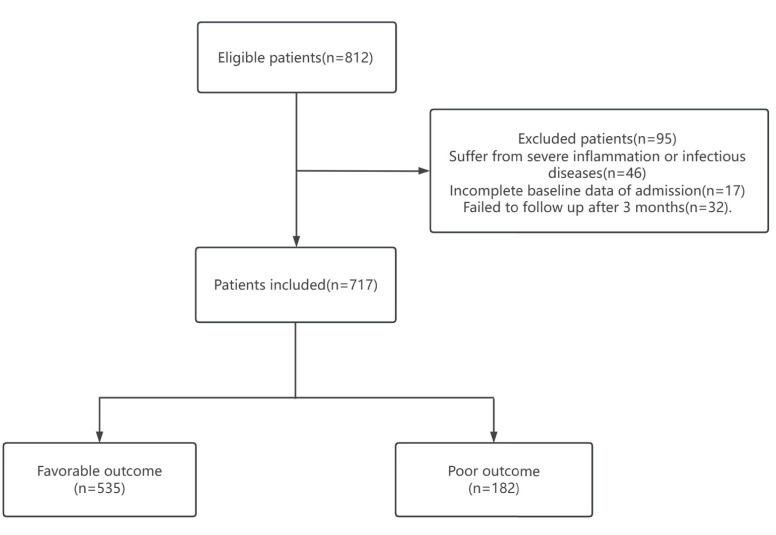
Flow diagram showing the patients selection process.

**Fig. (2):**
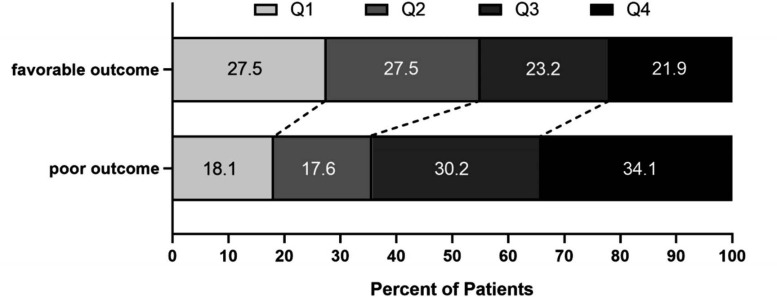
Distribution of PIV levels in the favorable outcome group and poor outcome group.

**Fig. (3):**
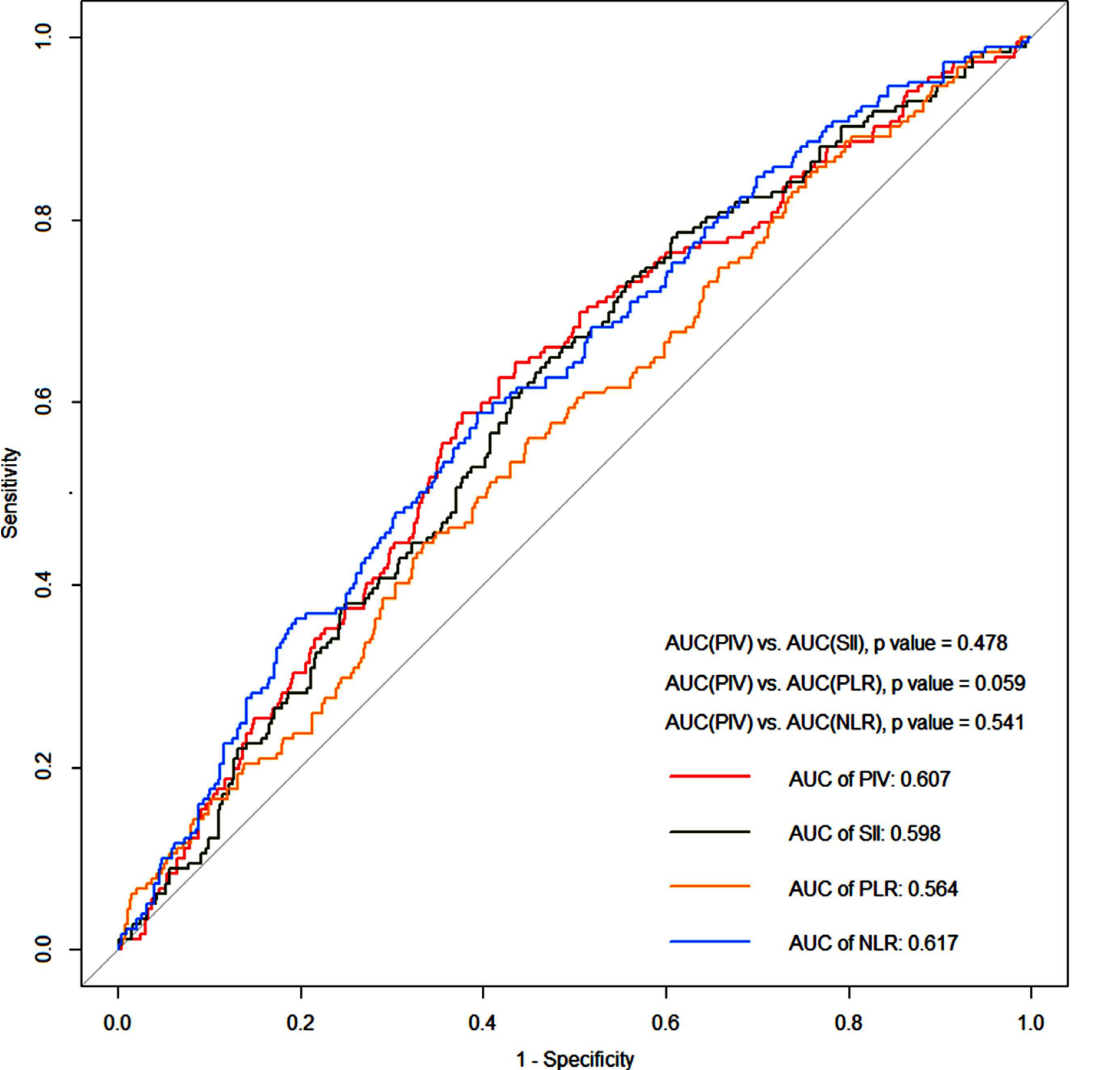
Receiver operating characteristic curves of PIV, PLR, NLR and SII on the prognosis of AIS patients treated with IVT.

**Table 1:** Demographics and clinical characteristics of the subgroup according to clinical outcomes.

**Variable**	**Total (n = 717)**	**Favorable Outcome Group (n = 535)**	**Poor Outcome Group (n = 182)**	***p***
**Demographics**				
Age (year)	68(58-75)	66(57-74)	73(65-80)	<0.001
Male n, (%)	485(67.6)	367(68.6)	118(64.8)	0.360
**Previous History n, (%)**				
Drink	131(18.3)	100(18.7)	31(17.0)	0.658
Smoke	188(26.2)	144(26.9)	44(24.2)	0.496
Hypertension	517(72.1)	379(70.8)	138(75.8)	0.214
Diabetes	188(26.2)	123(23.0)	65(35.7)	0.001
Atrial fibrillation	159(22.2)	93(17.4)	66(36.3)	<0.001
Anti-thrombotic	76(10.6)	51(9.5)	25(13.7)	0.125
Dyslipidemia	37(5.2)	27(5.0)	10(5.5)	0.847
History of stroke	127(17.7)	83(15.5)	44(24.2)	0.01
**Baseline Parameters**				
NIHSS score	6(3-11)	4(2-8)	12(8-18)	<0.001
SBP (mmHg)	157.57±24.8	158.22±24.7	155.64±25	0.255
DBP (mmHg)	88(79-99)	88(79-98)	89(78-100)	0.896
OTT, minute	180 (139-217)	177 (139-218)	182 (139-214)	0.559
Standard-dose rt-PA	174(24.3%)	129(24.1%)	45(24.7%)	0.738
**Laboratory Data**				
WBC, 10^9^ /L	7.59 (6.35-9.50)	7.47 (6.29-9.26)	7.98 (6.56-10.04)	0.034
Monocyte, 10^9^ /L	0.42 (0.33-0.52)	0.41 (0.32-0.51)	0.44 (0.33-0.55)	0.129
Platelet count, 10^9^ /L	196 (161-238)	198 (164-237)	186 (157-240)	0.164
Lymphocyte, 10^9^ /L	1.64 (1.20-2.23)	1.73 (1.25-2.27)	1.46 (1.05-1.90)	<0.001
Neutrophil, 10^9^ /L	5.11 (3.91-6.92)	4.91 (3.80-6.67)	5.86 (4.22-7.93)	<0.001
Glucose, mmol/L	6.98(5.84-8.89)	6.82(5.73-8.69)	7.48(6.23-10.24)	<0.001
TBIL	16.6(13.0-22.9)	16.4(12.6-21.8)	18.2(14.5-26.3)	<0.001
HDL, mmol/L	1.02(0.87-1.23)	1.01(0.86-1.20)	1.09(0.88-1.32)	0.009
LDL, mmol/L	2.71 (2.10-3.33)	2.72 (2.13-3.37)	2.69 (2.01-3.26)	0.370
TC, mmol/L	4.45 (3.80-5.11)	4.43 (3.83-5.10)	4.50 (3.68-5.20)	0.821
TG, mmol/L	1.26 (0.95-1.73)	1.29 (0.96-1.77)	1.14 (0.92-1.60)	0.008
PIV	244.21 (139.93-434.49)	223.80 (131.76-394.97)	316.32 (187.42-585.67)	<0.001
SII	605.1 (372.89-1004.59)	558.01 (345.60-907.26)	738.76 (488.59-1270.92)	<0.001
PLR	119.59 (88.79-169.33)	115.38 (84.82-167.60)	132.76 (96.65-173.35)	0.010
NLR	3.05 (1.96-5.22)	2.87 (1.81-4.76)	3.87 (2.39-6.56)	<0.001
**PIV n,(%) *p* <0.001**				
Q1 (<139.93)	180(25.1)	147(27.5)	33(18.1)	-
Q2 (139.93-244.21)	179(25)	147(27.5)	32(17.6)	-
Q3 (244.21-434.49)	179(25)	124(23.2)	55(30.2)	-
Q4 (>434.49)	179(25)	117(21.9)	62(34.1)	-
**TOAST Classification n, (%) *p* <0.001**				
LAA	329(45.9)	231(43.2)	98(53.8)	-
SAO	157(21.9)	145(27.1)	12(6.6)	-
CE	145(20.2)	83(15.5)	62(34.1)	-
(SOE+SUE)	86(12)	76(14.2)	10(5.5)	-

**Abbreviations:** SBP, systolic blood pressure; DBP, diastolic blood pressure; OTT, onset to treatment time; WBC, white blood cell count; TBIL, total bilirubin; HDL, high-density lipoprotein; LDL, low-density lipoprotein; TC, total cholesterol; TG, triglyceride; PIV, pan-immune-inflammation value; SII, systemic immune-inflammation index; PLR, platelet to lymphocyte ratio; TOAST, Trial of Org 10,172 in Acute Stroke Treatment; LAA, large artery atherosclerosis; SAO, small vessel occlusion; CE, cardioembolism; SOE, stroke of other determined etiology; SUE, stroke of undetermined etiology;

**Table 2:** Multivariate-adjusted odds ratios for clinical outcome stratified by PIV levels.

**Variables**	**Model 1**		**Model 2**		**Model 3**	
	**OR (95%CI)**	***p***	**OR (95% CI)**	***p***	**OR (95%CI)**	***p***
Age	1.048 (1.032-1.065)	<0.001	-	-	1.022 (1.001-1.043)	0.036
Gender (Male)	0.844 (0.592-1.204)	0.349	-	-	0.990 (0.626-1.564)	0.965
NIHSS score	1.214 (1.172-1.256)	<0.001	1.199 (1.158-1.242)	<0.001	1.200 (1.154-1.249)	<0.001
TBIL	1.034 (1.015-1.053)	<0.001	1.027 (1.008-1.047)	0.006	1.006 (0.982-1.029)	0.642
Glucose	1.061 (1.017-1.107)	0.007	1.060 (1.014-1.108)	0.010	0.995 (0.932-1.062)	0.869
Diabetes mellitus	1.861 (1.293-2.678)	0.001	1.841 (1.267-2.677)	0.001	3.333 (1.911-5.813)	<0.001
Atrial fibrillation	2.704 (1.857-3.938)	<0.001	1.952 (1.308-2.914)	0.001	0.840 (0.342-2.064)	0.704
TG	0.913 (0.754-1.107)	0.355	1.081 (0.906-1.290)	0.386	1.111 (0.905-1.363)	0.314
HDL	2.085 (1.230-3.536)	0.006	1.642 (0.956-2.821)	0.072	2.094 (1.038-4.224)	0.039
History of stroke	1.736 (1.150-2.622)	0.009	1.560 (1.023-2.379)	0.039	1.705 (1.029-2.825)	0.038
**TOAST Classification**						
LAA	-	<0.001	-	<0.001	-	0.006
SAO	0.195 (0.103-0.368)	<0.001	0.225 (0.119-0.428)	<0.001	0.293 (0.144-0.596)	0.001
CE	1.761 (1.174-2.64)	0.006	1.511 (0.992-2.300)	0.054	1.015 (0.412-2.502)	0.974
(SOE+SUE)	0.310 (0.154-0.625)	0.001	0.371 (0.182-0.756)	0.006	0.606 (0.273-1.343)	0.217
**PIV Quartiles**						
Q1 (<139.93)	-	<0.001	-	<0.001	-	0.020
Q2 (139.93-244.21)	0.970 (0.567-1.660)	0.911	1.029 (0.595-1.781)	0.918	1.134 (0.595-2.163)	0.702
Q3 (244.21-434.49)	1.976 (1.206-3.236)	0.007	2.239 (1.345-3.725)	0.002	1.905 (1.040-3.489)	0.037
Q4 (>434.49)	2.361 (1.450-3.842)	0.001	2.655 (1.603-4.395)	<0.001	2.229 (1.229-4.044)	0.008

**Notes:** Model 1 is a univariate analysis. Model 2 is adjusted for age and sex. Model 3 is adjusted for age, sex, NIHSS score, TBIL, glucose, diabetes mellitus, atrial fibrillation, history of stroke, TG, HDL, and TOAST classification.

**Table 3:** Diagnostic values of the PIV, SII, PLR, NLR for poor outcome in AIS patients after IVT.

**-**	**AUC (95% CI)**	**Sensitivity (%)**	**Specificity (%)**	**Cutoff Value**	***p***	p*
PIV	0.607 (0.560-0.654)	59	62	283.84	0.000	-
SII	0.598(0.552-0.645)	65	53	582.755	0.000	0.478
PLR	0.564(0.516-0.612)	56	55	124.02	0.010	0.059
NLR	0.617(0.570-0.663)	59	60	3.425	0.000	0.541

**Note: **p* for comparison of AUC between groups; **Abbreviations:** AUC, area under the curve; CI, confidence interval; PIV, pan-immune-inflammation value; SII, systemic immune-inflammation index; PLR, platelet to lymphocyte ratio; NLR, neutrophil to lymphocyte ratio.

## Data Availability

The data that support the findings of this study are available on request from the corresponding author.

## LIST OF ABBREVIATIONS

mCRC: Metastatic Colorectal Cancer
NIHSS: National Institute of Health Stroke Scale
OTT: Onset To Treatment
ROC: Receiver Operating Curve
TOAST: Trial of Org 10172 in Acute Stroke Treatment
VIF: Variance Inflation Factors

## References

[1] Feigin V.L., Stark B.A., Johnson C.O. (2021). *et al.*Global, regional, and national burden of stroke and its risk factors, 1990–2019: A systematic analysis for the Global Burden of Disease Study 2019.. Lancet Neurol..

[2] Feigin V.L., Norrving B., Mensah G.A. (2017). Global burden of stroke.. Circ. Res..

[3] Endres M., Moro M.A., Nolte C.H., Dames C., Buckwalter M.S., Meisel A. (2022). Immune pathways in etiology, acute phase, and chronic sequelae of ischemic stroke.. Circ. Res..

[4] Weng Y., Zeng T., Huang H. (2021). *et al.*Systemic immune-inflammation index predicts 3-month functional outcome in acute ischemic stroke patients treated with intravenous thrombolysis.. Clin. Interv. Aging.

[5] Gong P., Liu Y., Gong Y. (2021). *et al.*The association of neutrophil to lymphocyte ratio, platelet to lymphocyte ratio, and lymphocyte to monocyte ratio with post-thrombolysis early neurological outcomes in patients with acute ischemic stroke.. J. Neuroinflammation.

[6] Fucà G., Guarini V., Antoniotti C. (2020). *et al.*The pan-immune-inflammation value is a new prognostic biomarker in metastatic colorectal cancer: Results from a pooled-analysis of the valentino and TRIBE first-line trials.. Br. J. Cancer.

[7] Murat B., Murat S., Ozgeyik M., Bilgin M. (2023). Comparison of pan‐immune‐inflammation value with other inflammation markers of long‐term survival after ST ‐segment elevation myocardial infarction.. Eur. J. Clin. Invest..

[8] Tong X., Wang C., Liao X. (2016). *et al.*Smoking–thrombolysis relationship depends on ischemic stroke subtype.. Stroke.

[9] Zhong C., Lv L., Liu C. (2014). *et al.*High homocysteine and blood pressure related to poor outcome of acute ischemia stroke in Chinese population.. PLoS One.

[10] Wang Y., Cui L., Ji X. (2011). *et al.*The China National Stroke Registry for patients with acute cerebrovascular events: design, rationale, and baseline patient characteristics.. Int. J. Stroke.

[11] Luo Y., Jin H., Guo Z.N. (2019). *et al.*Effect of hyperhomocysteinemia on clinical outcome and hemorrhagic transformation after thrombolysis in ischemic stroke patients.. Front. Neurol..

[12] Kopin L., Lowenstein C.J. (2017). Dyslipidemia.. Ann. Intern. Med..

[13] DeLong J.H., Ohashi S.N., O’Connor K.C., Sansing L.H. (2022). Inflammatory responses after ischemic stroke.. Semin. Immunopathol..

[14] Wu F., Liu Z., Zhou L. (2022). *et al.*Systemic immune responses after ischemic stroke: From the center to the periphery.. Front. Immunol..

[15] Tian X., Wang P., Chen S. (2023). *et al.*Association of serum uric acid to lymphocyte ratio, a novel inflammatory biomarker, with risk of stroke: A prospective cohort study.. CNS Neurosci. Ther..

[16] Zhang X.G., Xue J., Yang W.H. (2021). *et al.*Inflammatory markers as independent predictors for stroke outcomes.. Brain Behav..

[17] Liu Y.L., Wu Z.Q., Qu J.F. (2020). *et al.*High neutrophil‐to‐lymphocyte ratio is a predictor of poor short‐term outcome in patients with mild acute ischemic stroke receiving intravenous thrombolysis.. Brain Behav..

[18] Wang N., Yang Y., Qiu B. (2022). *et al.*Correlation of the systemic immune-inflammation index with short- and long-term prognosis after acute ischemic stroke.. Aging.

[19] Xu J.H., He X.W., Li Q. (2019). *et al.*Higher platelet-to-lymphocyte ratio is associated with worse outcomes after intravenous thrombolysis in acute ischaemic stroke.. Front. Neurol..

[20] Guven D.C., Sahin T.K., Erul E., Kilickap S., Gambichler T., Aksoy S. (2022). The association between the pan-immune-inflammation value and cancer prognosis: A systematic review and meta-analysis.. Cancers.

[21] Ministrini S., Carbone F., Montecucco F. (2021). Updating concepts on atherosclerotic inflammation: From pathophysiology to treatment.. Eur. J. Clin. Invest..

[22] Cao X., Zhu Q., Xia X. (2020). *et al.*The correlation between novel peripheral blood cell ratios and 90-day mortality in patients with acute ischemic stroke.. PLoS One.

[23] Chen Y., Ren J., Yang N. (2021). *et al.*Eosinophil-to-monocyte ratio is a potential predictor of prognosis in acute ischemic stroke patients after intravenous thrombolysis.. Clin. Interv. Aging.

[24] Sun Y.Y., Wang M.Q., Wang Y. (2022). *et al.*Platelet-to-lymphocyte ratio at 24h after thrombolysis is a prognostic marker in acute ischemic stroke patients.. Front. Immunol..

[25] Meng J., Zhang J., Fang J. (2022). *et al.*Dynamic inflammatory changes of the neurovascular units after ischemic stroke.. Brain Res. Bull..

[26] Wang J., Jiang Y., Zeng D., Zhou W., Hong X. (2020). Prognostic value of plasma HMGB1 in ischemic stroke patients with cerebral ischemia-reperfusion injury after intravenous thrombolysis.. J. Stroke Cerebrovasc. Dis..

[27] Chen H., Luan X., Zhao K. (2018). *et al.*The association between neutrophil-to-lymphocyte ratio and post-stroke depression.. Clin. Chim. Acta.

[28] Huang G., Chen H., Wang Q. (2019). *et al.*High platelet-to-lymphocyte ratio are associated with post-stroke depression.. J. Affect. Disord..

[29] Yan D., Dai C., Xu R., Huang Q., Ren W. (2023). Predictive ability of systemic inflammation response index for the risk of pneumonia in patients with acute ischemic stroke.. Gerontology.

